# Integrative population genomics and tissue-specific expression profiling in cattle using whole-genome sequence resources

**DOI:** 10.1186/s12864-026-13218-4

**Published:** 2026-07-25

**Authors:** Mohammed Saeed-Zidane, Iulia Blaj, Amira Yousif, Georg Thaller

**Affiliations:** Molecular Genetics Group, Institute of Animal Breeding and Husbandry, Christian-Albrechts-University Kiel, Kiel, Germany

**Keywords:** Population genomics, Whole-genome sequencing, NF-κB signaling, Innate immunity, Bovine

## Abstract

Large-scale genomic resources enable systematic investigation of genetic variation and tissue-specific expression of immune-related pathways in livestock species. Here, we integrated population-level whole-genome sequence data with tissue-specific transcriptional profiling to characterize genomic differentiation and expression patterns of NF-κB pathway-related genes in cattle. Using whole-genome sequence data from the 1000 Bull Genomes Project, we identified prioritized differentiated variants within several NF-κB pathway–related genes, including *CD14*, *NF-κB1*, *IL-1β*, and *BAFFR*. The mRNA expression levels of selected NF-κB signaling-related genes were quantified across four tissues with relevance to host defense and metabolism in bulls and cows of both breeds. Expression analyses revealed pronounced tissue-dependent regulation, with distinct organ-specific transcriptional patterns across the investigated genes. Intestinal tissues showed lower expression of several innate immune genes, whereas *CD14* expression was more prominent in liver-associated comparisons, highlighting functional tissue specialization within the NF-κB signaling network. Breed- and sex-associated effects were gene-dependent rather than uniform across the pathway. Histological assessment of the spleen was performed to provide an anatomical context for observed transcriptional variations. A population-prioritized *CD14* missense variant (Asn177Asp), identified from the 1000 Bull Genomes dataset, was contextualized using in silico structural annotation to provide structural context for coding variation. These findings provide descriptive evidence that population-level genomic differentiation in selected NF-κB pathway genes is accompanied by tissue-specific transcriptional differences in cattle.

**Supplementary Information:**

The online version contains supplementary material available at 10.1186/s12864-026-13218-4.

## Introduction

NF-κB signaling plays a central role in bovine innate and adaptive immunity and comprises transcription factors that regulate gene networks involved in immune responses [1], mediating both innate and adaptive immunity [2]. Several pathways lead to *NF-κB1* activation. On the one hand, the canonical pathway is induced by tumor necrosis factor-alpha (*TNF-α*), interleukin-1 (*IL-1β*) proteins, or byproducts of bacterial and viral infections [3]. On the other hand, the noncanonical pathway is triggered by particular members of the tumor necrosis factor superfamily, such as the *BAFF* protein [4]. NF-κB1 protein has been widely implicated in immune cell development and lymphoid tissue biology [2]. Variation within NF-κB pathway genes provides an opportunity to study how genomic diversity is reflected in tissue-specific transcriptional profiles [3]. The activation of immune responses and signaling pathways is influenced by host genetics, sex, age, history of vaccination, prior infection, nutritional status, physiological and/or environmental stressors, and management [5, 6]. Host genetics is a main factor involved in immunocompetence differences between and within breeds. Therefore, numerous studies have been conducted to investigate genetic markers associated with immune-related phenotypic variation [5, 6].

Single-nucleotide polymorphisms (SNPs) represent the most common form of genetic variation [7] and have been associated with susceptibility to infectious and inflammatory diseases [8, 9]. In cattle, SNPs within immune-related genes, including Toll-like receptors and cytokines, have been linked to variation in immune-related traits [10, 11]. Furthermore, Seabury et al. (2010) [12] reported that 220 diallelic variants were found, with an average of one locus per 219 bp in bovine innate immune genes (*TLR1* and *TLR10*). Other studies have demonstrated that the protein of the *IL6* gene is a marker involved in the pathogenesis of most studied inflammatory diseases [13]. Two presumed functional SNPs were detected within the upstream 100 bp region of exon 2 in bovine *IL6* [14]. Moreover, Wang et al. (2014) [15] reported that the SNP located in intron 8 of the bovine *CD46* gene was associated with partial retention of the intron 8 sequence within the mature RNA. However, further studies have reported that certain SNPs that are correlated with mastitis, ketosis, and metritis need to be functionally investigated [11, 16–18]. Furthermore, there are breed-dependent SNPs involved in immune response differences among cattle breeds [19].

Despite extensive functional characterization of NF-κB signaling, integrative studies linking population-scale genomic differentiation with tissue-specific transcriptional patterns remain limited in cattle. Integrating population-scale genomic variation with experimental transcriptional profiling provides a functional framework for prioritizing candidate loci associated with breed-specific immune traits. In this study, variants identified from large-scale cattle genomic resources were used to prioritize immune-related genes for downstream expression analysis in experimentally sampled Holstein and Charolais cattle, enabling functional interpretation of genomic differentiation across immune-relevant tissues.

## Materials and methods

### Genomic data sources and tissue sampling

Two different bovine breeds for production purposes, dairy (Holstein) and beef (Charolais) cattle, were selected based on the availability of genomic resources from the 1000 Bull Genomes Project, and their relevance within German and international cattle production systems. The study combined population-scale genomic data with targeted experimental tissue-based expression analyses. Publicly available whole-genome variant data from the 1000 Bull Genomes Project (Run 7) [20], comprising 844 Holstein and 144 Charolais animals, were used for population-level variant prioritization in candidate immune genes. For experimental analyses, tissue samples were collected from 16 slaughtered cattle (4 bulls and 4 cows per breed). These animals were selected based on slaughterhouse records for breed assignment, health status, and production background. The average age ranged from 18 to 20 months for bulls and 24 to 30 months for cows. A subset of eight animals (4 Holstein bulls and 4 Charolais bulls) was additionally used for targeted validation of prioritized genomic variants in candidate immune gene regions. All ethics related to sample collection and animal handling were conducted according to the approved protocols and procedures of Vion Bad Bramstedt GmbH (Germany). All animals originated from routine commercial slaughter and were not subjected to experimental infection, immune challenge, or pharmacological intervention before sampling. To ensure normal health status, external and internal organ morphology was assessed in consultation with slaughterhouse veterinarians before sample collection. Only animals without clinical signs of disease or organ abnormalities were included. Upon slaughtering, parts of the spleen, intestine, lung, and liver were precisely collected. These organs were considered in this study based on their immunological roles [21–24], accessibility to these organs within the slaughterhouse workflow, and agreement with the slaughterhouse representatives. Accordingly, the collected samples were immersed in physiological saline (NaCl), followed by two washes with phosphate-buffered saline (PBS). Samples intended for DNA analysis were obtained from liver tissue. Independent tissue sections from spleen, intestine, lung, and liver were collected for RNA analysis. Additional spleen samples were fixed immediately in 4% paraformaldehyde for histological assessment. All samples were transported on dry ice. Candidate genes related to NF-κB signaling were selected based on their biological roles in canonical and noncanonical immune signaling pathways, including receptors (*CD14*, *TLR4*, *BAFFR*), ligands and pro-inflammatory mediators (*TNF-α*, *IL-1β*, *BAFF*), and the transcription factor *NF-κB1*, together with preliminary population-genomic differentiation observed in the 1000 Bull Genomes dataset.

### Breed-specific gene sequence-based investigations using 1000 Bull Genomes Project data

A total of 988 individuals (844 Holstein and 144 Charolais) with whole-genome sequence data, including SNPs and small indels, were selected from the 1000 Bull Genomes Project (Run 7) [20]. Variant information was extracted from the available variant calling format (VCF) file for genomic regions spanning ± 1000 bp from the annotated start and end positions of the following candidate genes: *CD14*,* TLR4*,* NF-κB1*,* TNF-α*,* IL-1β*,* BAFF*, and *BAFFR*. Gene coordinates were based on Ensembl release 109 [25]. Variant consequences were predicted using the Ensembl Variant Effect Predictor (VEP) Web Interface [26]. VCFTools v0.1.16 was used for variant processing and calculation of population differentiation statistics. Genetic differentiation between breeds was quantified using Weir and Cockerham’s fixation index (FST) [27], as implemented in VCFTools [28]. To prioritize variants showing near-complete differentiation within the analyzed gene set, a stringent within-gene FST threshold (≥ 0.95) was applied as a pre-specified internal ranking rule within each gene to select relative outliers for downstream annotation and validation. This threshold was not and should not be seen or interpreted as a genome-wide measure of divergence, evidence of selection, or statistical significance. Variants meeting this criterion were extracted from the VEP output to assess their predicted effects on genes, transcripts, protein sequences, and regulatory regions, and those with the most severe predicted impacts were selected for validation in experimental animals. To assess the genetic positioning of the experimentally sequenced validation animals (4 Holstein and 4 Charolais bulls) relative to the broader breed populations, principal component analysis (PCA) was performed using merged variant calls from the seven candidate immune genes across these experimental animals and corresponding 1000 Bull Genomes reference samples. Because only targeted regional variant data were available for the experimentally sequenced animals, PCA was restricted to the candidate-gene regions rather than performed genome-wide. The reads were aligned using bwa v0.7.17 [29] to the RefSeq *Bos Taurus* reference genome assembly ARS-UCD2.0 from NCBI (*GCF_002263795.3_ARS-UCD2.0_genomic.fna*). Regional bam files corresponding to the genes of interest were extracted. Statistics on the regional bam files were calculated with samtools v1.21 [30] with the samtools depth command. Multisample variant calling was performed using BCFtools v1.18 [30] with the commands bcftools mpileup and bcftools call. Raw calls were filtered using QUAL$\:\:\ge\:\:$20, and total site depth$\:\:\ge\:\:$10. The PCA analysis was performed with plink2 v2.0.0 [31] on biallelic SNPs, and the PCA plot was generated with R v4.5.2 [32].

### DNA isolation, genome sequencing, and variant calling

Genomic DNA was extracted via a DNeasy Blood and Tissue Kit (Qiagen, Germany). Briefly, 25 mg of liver tissue (two samples/animal) was subjected to lysis via animal tissue lysis buffer supplemented with proteinase K, after which the column-based protocol was used according to the manufacturer’s instructions. The purified DNA was stored at -80 °C until its shipment on dry ice for sequencing at BGI (BGI GmbH, China). DNA quality control, including concentration, sample integrity, and purity, was assessed via BGI. The DNA concentration was measured with a fluorometer (Qubit Fluorometer, Invitrogen, Germany). Moreover, DNA integrity and purity were assessed by agarose gel electrophoresis on a 1% agarose gel at 100 V for 40 min. High-quality DNA samples were selected for library preparation according to the BGI library preparation pipeline, in which 300–500 bp inserts were used for paired-end libraries. Ultimately, sequencing (30X/sample) was conducted on the DNBSEQ platform to generate 100 bp paired-end reads. Sequencing quality metrics for the experimentally sequenced validation animals, including coverage and read-depth statistics across candidate gene regions, are provided in Supplementary Table 2.

### RNA extraction and cDNA synthesis

Total RNA was extracted from the collected tissues via a miRNeasy Mini Kit (Qiagen, Hilden, Germany) according to the manufacturer’s instructions. The RNA concentration was measured via a NanoDrop^®^ ND-1000 Spectrophotometer (PEQLAB Biotechnologie GmbH, Germany). cDNA was synthesized from total RNA via a first-strand cDNA synthesis kit (Thermo Fisher Scientific, Germany). Briefly, the RNA concentration was adjusted via RNase-free water, and a maximum volume of 10 µl of adjusted RNA was co-incubated with 0.5 µl of 100 µM oligo (dT)_18_ and 0.5 µl of random primer at 65 °C for 5 min. Afterward, 1 µl of RiboLock RNase Inhibitor, 4 µl of 5x Reaction Buffer, 2 µl of dNTPs, and 2 µl of RevertAid Reverse Transcriptase were added and incubated at 25 °C for 5 min and 37 °C for 60 min, followed by 70 °C for 5 min. Then, the cDNA samples were kept at -20 °C until use in the gene expression analysis.

### mRNA expression analysis

The relative mRNA expression levels of the candidate genes involved in the NF-κB signaling pathway (*CD14*, *TLR4*, *NF-κB1*, *TNF-α*, *IL-1β*, *BAFF*, and *BAFFR*) were quantified via quantitative PCR (qPCR) in a Roche 1.1 System (Roche, Germany). qPCR was performed using the synthesized cDNA and iTaq™ Universal SYBR^®^ Green Supermix (Bio-Rad Laboratories GmbH, Germany). The qPCR procedure involved the following program: 95 °C for 3 min; 40 cycles of 95 °C for 15 s and 60 °C for 45 s; and melting curve analysis. For qPCR normalization, Ct values of target genes were normalized against the mean of three constitutively expressed reference genes (*GAPDH*, *YWHAZ*, and *B2M*). Normalized delta Ct (ΔCt) values were used for statistical inference, whereas relative expression values shown in figures were calculated using the comparative threshold cycle (2^-ΔΔCt) method [33, 34]. All primers (Supplementary Table 1) were designed via the Primer3 online tool.

### Histological context of splenic tissue

Histological analysis focused on the spleen because of its immunological relevance. For spleen histological analysis, some of the washed spleen samples were fixed directly at the slaughterhouse in 4% paraformaldehyde. The fixed samples were washed well with running water before dehydrating with ascending grades of ethanol overnight in 70% ethanol. Thereafter, the samples were submerged twice in methyl benzoate and subsequently immersed overnight in methyl benzoate before being embedded in paraffin, followed by standard dehydration and clearing procedures. Thereafter, the samples were subjected to routine hematoxylin and eosin (H&E) staining. Finally, the slides were examined under a Keyence BZ-X800 microscope (Keyence, Japan). The histomorphometry analyses were performed using custom-written Python (v3.14.0). Identical preprocessing, segmentation, and measurement parameters were applied uniformly to all samples. Image preprocessing and tissue segmentation were implemented using the OpenCV library (v4.13.0), while color deconvolution, threshold-based segmentation, and morphometric measurements were performed using scikit-image (v0.26.0). Segmentation outputs were visually inspected on overlay images to confirm appropriate identification of splenic compartments. Histology was included as contextual phenotyping and was not used to infer pathway function.

### Optional in silico structural contextualization of coding variation

The three-dimensional structure of bovine *CD14* was obtained from the Protein Data Bank and used as the wild-type model. The N177D variant was generated by substituting asparagine with aspartic acid at position 177 in chain A. Protein structures were prepared by removing non-protein components and ensuring compatibility with downstream analyses. The effect of the mutation on protein stability was evaluated using DynaMut2, which predicts changes in folding free energy (*ΔΔG*) between the wild-type and mutant structures. To assess mutation-induced changes in protein dynamics, flexibility simulations were conducted using the CABS-flex 2 server. Residue-level backbone fluctuations were quantified as root-mean-square fluctuation (RMSF) values and compared between the two systems. Molecular docking was performed using the CB-Dock2 web server, which combines automated cavity detection with AutoDock Vina. Blind docking was performed for both wild-type and N177D *CD14*, using lipid A/MPLA (Monophosphoryl lipid A*)* headgroup (pubchem.ncbi.nlm.nih.gov), separately using default parameters. The top-ranked binding pockets and poses were identified based on predicted binding affinities.

### Statistical analysis

Gene expression data were analyzed using delta Ct (ΔCt) values, treated as the dependent variable for inference because they better preserve the scale assumptions required for linear modeling of qPCR data. Relative expression (fold change; 2^-ΔΔCt) values were used only for graphical presentation. For each candidate gene, a factorial general linear model (Gaussian error structure) was fitted with breed, sex, and tissue as fixed effects, including all interaction terms (breed × sex, breed × tissue, sex × tissue, and breed × sex × tissue). Statistical analyses were performed in Jamovi (version 2.7.28), and post hoc multiple comparisons were conducted using Tukey’s adjustment where appropriate. Model assumptions were evaluated using Shapiro–Wilk tests of residual normality, Levene’s test for homogeneity of variance, and visual inspection of Q–Q plots. Statistical significance was defined as *p* ≤ 0.05. Figures were generated in GraphPad Prism (version 9.1.1), with expression values displayed as fold change (2^-ΔΔCt) and presented as mean ± SEM. Histomorphometric spleen measurements were analyzed separately using two-way ANOVA with breed and sex as fixed factors, including the breed × sex interaction. When significant effects were detected, Tukey’s post hoc test was used for pairwise comparisons.

### Study design considerations

This study was designed as an integrative, descriptive analysis combining population-level genomic data with targeted tissue-specific profiling. Methodological choices, including within-gene FST outlier ranking, targeted qPCR rather than transcriptome-wide sequencing, and illustrative in silico structural annotation, were selected to support genomic observations while maintaining feasibility and reproducibility. These methodological choices supported genomic observations through targeted tissue-specific transcription profiling and were not intended to provide functional validation.

## Results

### Prioritizing variants based on the 1000 Bull Genomes Project data

To identify breed-associated genetic differentiation within NF-κB signaling-related genes, we first analyzed whole-genome sequence variation using population-level data. To assess the putative functional impact of the Holstein and Charolais variants harbored by the genes of interest, the Variant Effect Predictor tool was utilized. In terms of the predicted molecular impacts of the genomic variants, the majority were classified as intron variants, except for CD14 and TNF-α, where the majority were classified as intergenic variants. A summary of the inferred impacts and their percentage breakdowns is presented in Table 1. The F_ST_ statistic, which quantifies allele frequency differentiation between the Holstein and Charolais breeds, was calculated either per SNP (SNP F_ST_) or by using a sliding window approach (window F_ST_). Because negative values hold no biological interpretation, they were set to zero before summarization. The mean F_ST_ values per gene for the two different scenarios and descriptive statistics are available in Table 2.

**Table 1:** Percentage (%) breakdown of the variant consequences as inferred by VEP per gene

Consequence	Gene Symbol						
	*CD14*	*TLR4*	*NF-κB1*	*TNF-α*	*IL-1β*	*BAFF*	*BAFFR*
3_prime_UTR_variant	23.256	2.685	0.781	11.475	9.091	1.175	19.512
5_prime_UTR_variant	2.326	2.013	0	0	7.273	0.94	0
inframe_deletion	2.326	0	0	0	0	0	0
inframe_insertion	2.326	0	0	0	0	0	0
intergenic_variant	58.139	20.134	0.26	44.262	19.091	2.9	29.268
intron_variant	2.325	64.429	97.344	31.148	60.909	94.436	36.585
missense_variant	4.651	4.027	0.625	1.639	0.909	0.235	4.878
splice_acceptor_variant	0	0	0.104	0	0	0	0
splice_donor_region_ variant, intron_variant	0	0	0	0	0.909	0	0
splice_polypyrimidine_ tract_variant, intron_variant	0	0	0.312	0	0	0	2.439
splice_region_variant, intron_variant	0	0	0.052	0	0	0.157	0
splice_region_variant, splice_polypyrimidine_ tract_variant, intron_variant	0	0	0.104	0	0	0	2.439
splice_region_variant, synonymous_variant	0	0	0	1.639	0	0	0
synonymous_variant	4.651	6.711	0.417	9.836	1.818	0.157	4.878
Total variants	43	149	1920	61	110	1276	41

**Table 2:** Descriptive statistics of the candidate genes and mean values for the F_ST_ statistic (SNP and window approach). CHR refers to chromosomes, and bp refers to base pairs

Gene Symbol	CHR	Start (bp)	End (bp)	Length (bp)	Number of SNPs	Mean SNP Fst	Mean window Fst
*CD14*	7	51,762,895	51,765,768	2873	52	0.0155	0.0138
*TLR4*	8	107,057,826	107,068,836	11,010	241	0.0095	0.0323
*NF-κB1*	6	22,205,847	22,328,613	122,766	2469	0.0193	0.0721
*TNF-α*	23	27,716,168	27,719,047	2879	104	0.0114	0.0147
*IL-1β*	11	46,543,394	46,552,101	8707	183	0.0549	0.1932
*BAFF*	12	83,641,466	83,672,595	31,129	853	0.0221	0.0711
*BAFFR*	5	112,905,857	112,907,803	1946	51	0.0212	0.0213

For the window approach, the weighted F_ST_ was considered. The mean SNP F_ST_ values varied from 0.0095 (*TLR4*) to 0.0549 (*IL-1β*), whereas the mean window F_ST_ values ranged from 0.0138 (*CD14*) to 0.1932 (*IL-1β*). Variants meeting the within-gene F_ST_ outlier criterion were selected for downstream annotation and validation. The impacts of preselected variants on all the genes were categorized as follows: three 3’ UTR variants (1.67%), two 5’ UTR variants (1.11%), six intergenic variants (3.33%), one hundred sixty-three intron variants (90.55%), two missense variants (1.11%), and four synonymous variants (2.22%). While the majority of the variants were modifier types (i.e., predictions were difficult or there was no evidence of impact), one missense variant and two synonymous variants had moderate (might change protein effectiveness) and low (unlikely to change protein) impacts, respectively. These variants were also investigated in additional experimental animals, for which the genotypes and allele frequencies are reported in Table 3.

**Table 3:** Genotypes and allele frequencies of the eight experimental animals from the Charolais (C) and Holstein (H) breeds and the cohort (1000 Genome Project) samples for the strongly differentiated variants. Freq Ref refers to the frequency reference, and Freq Alt refers to the frequency alternative

Location	Allele	Consequence	Gene	Breed	Genotype	Freq Ref	Freq Alt	Freq Ref Cohort	Freq Alt Cohort
5:112907278–112,907,278	T	synonymous variant	*BAFFR*	Charolais	C/C	1	0	0.937	0.063
				Holstein	C/C	1	0	0.996	0.004
7:51765080–51,765,080	C	missense variant	*CD14*	Charolais	T/C	0.875	0.125	0.840	0.160
				Holstein	T/C	0.875	0.125	0.931	0.069
6:22225005–22,225,005	A	synonymous variant	*NF-κB1*	Charolais	G/A	0.625	0.375	0.816	0.184
				Holstein	G/G	1	0	1	0

To further assess whether the sequenced validation animals were genetically consistent with their declared breed backgrounds, principal component analysis was performed using merged variant data from the seven candidate immune genes together with corresponding 1000 Bull Genomes reference samples (Supplementary Figure S1). The resulting clustering was broadly consistent with declared breed assignment within the candidate-gene variant space analyzed. Supplementary Table 2 provides per-sample and per-gene sample information related to the sequencing data (i.e., regional bam) of the experimental animals.

### Transcriptional abundance of the NF-κB signaling candidate genes

**Fig. 1:**
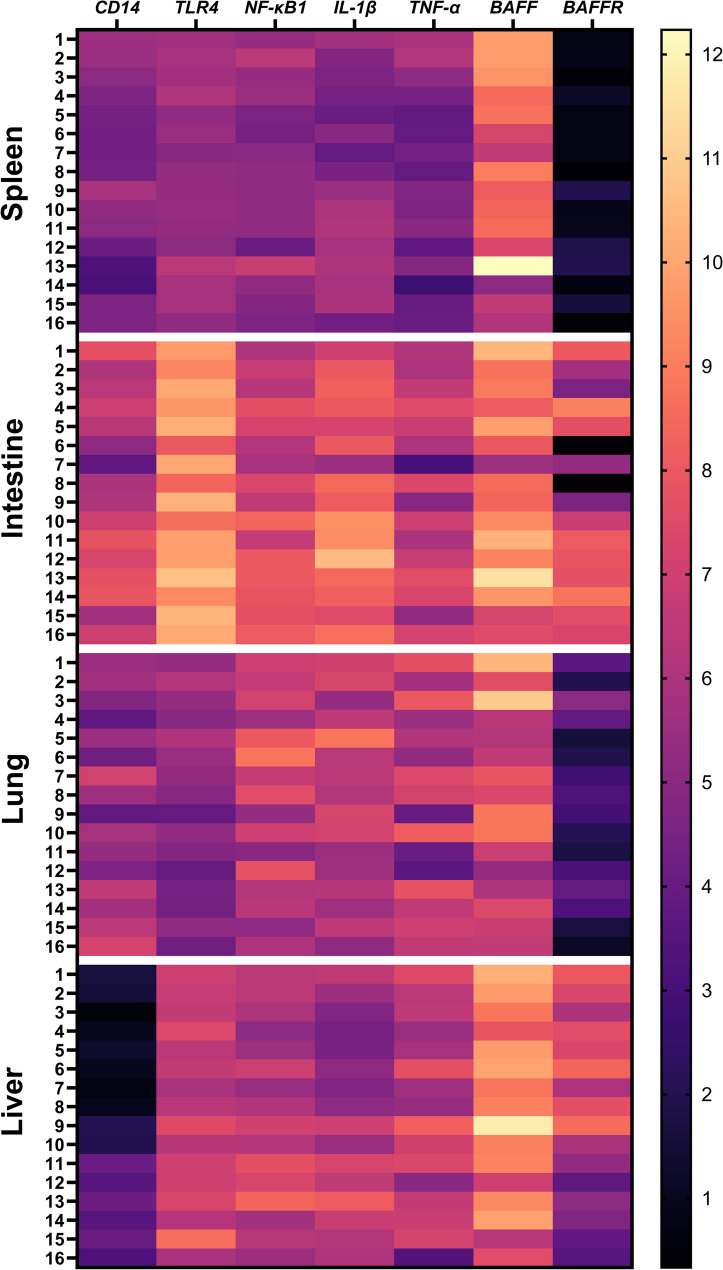
Heatmap visualization of tissue-specific relative abundance of NF-κB pathway-related genes across individual experimental animals. Transcript abundance expressed as ΔCt values for *CD14*, *TLR4*, *NF-κB1*, *IL-1β*, *TNF-α*, *BAFF*, and *BAFFR* across spleen, intestine, lung, and liver tissues from Holstein bulls (1–4), Charolais bulls (5–8), Holstein cows (9–12), and Charolais cows(13–16). Darker color indicates lower ΔCt values, which correspond to higher relative transcript abundance

Gene abundance profiling revealed clear tissue- and gene-specific expression patterns across the studied animal groups. This visualization is consistent with the ΔCt-based analysis, where darker color reflects lower ΔCt, indicating higher expression. Overall, tissue type appeared to be the dominant determinant of transcript abundance, with additional variability attributable to breed- and sex-associated differences for selected genes. Except for the *CD14* gene in the liver, the spleen showed high abundance for most investigated genes compared with other tissues. *BAFFR* was the most abundant transcript in the spleen and lung, whereas *CD14* was abundant in the liver (Fig. 1).

### Expression of canonical NF-κB signaling receptor genes

**Fig. 2:**
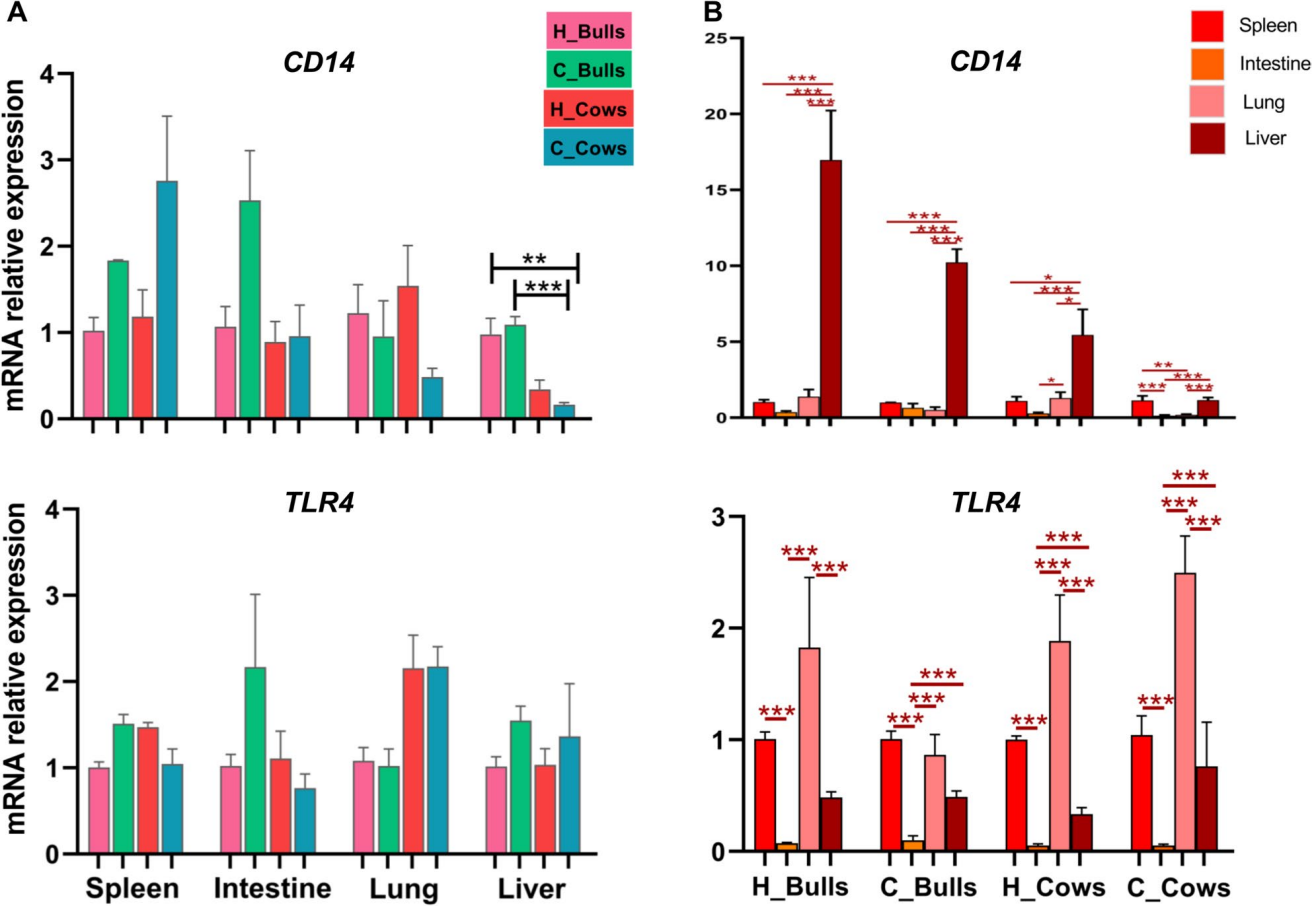
The mRNA expression levels of cell receptor genes (*CD14* and *TLR4*) in multiple comparisons between bulls and cows of the Charolais and Holstein breeds (**A**) and between different organs within each experimental animal group (**B**). The data are presented as the means ± SEMs from four independent biological replicates per group (each replicate corresponds to one individual animal). Stars indicate statistically significant Tukey-adjusted post hoc differences; * (*p* < 0.05), ** (*p* < 0.01), *** (*p* < 0.001), and **** (*p* < 0.0001). H_ refers to Holstein cattle, and C_ refers to Charolais cattle. Statistical analyses were performed using ΔCt values within a factorial general linear model including breed, sex, tissue, and their interactions. Relative expression values are presented as fold change for visualization

Analysis of variance revealed distinct expression patterns among the investigated immune-related genes. *CD14* expression was significantly affected by sex (F = 19.297, *p* < 0.001) and tissue (F = 91.398, *p* < 0.001), with significant breed × sex (F = 4.313, *p* = 0.043), breed × tissue (F = 6.500, *p* < 0.001), and sex × tissue (F = 7.543, *p* < 0.001) interactions, whereas breed alone and the three-way interaction were not significant. Post hoc analysis showed marked tissue-specific variation, with higher *CD14* expression in the liver compared with the spleen, intestine, and lung. In the liver, bulls of both breeds showed significantly higher expression levels compared with cows, while Holstein cows also showed significant tissue-specific differences involving the liver (Fig. 2). For *TLR4*, expression was significantly influenced by tissue (F = 193.047, *p* < 0.001), with significant breed × sex (F = 6.071, *p* = 0.017) and sex × tissue (F = 4.600, *p* = 0.007) interactions, whereas breed, sex, breed × tissue, and the three-way interaction were not significant. Post hoc comparisons revealed a highly consistent tissue-dependent pattern, with intestinal tissues generally showing lower *TLR4* expression compared with spleen, lung, and liver across all animal groups (Fig. 2).

### Differential expression levels of the NF-κB1 gene and its downstream pro-inflammatory genes

**Fig. 3:**
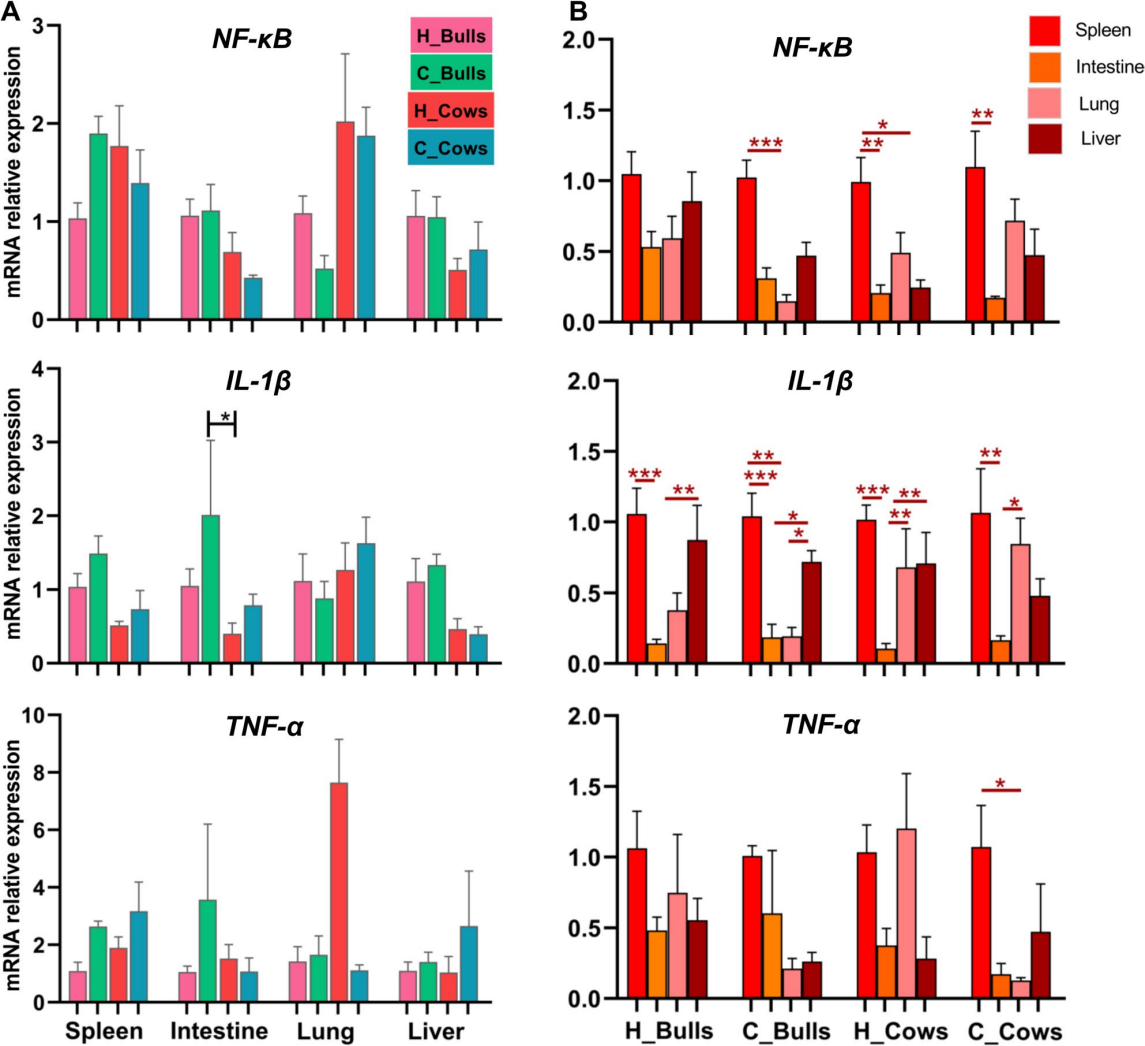
Relative mRNA expression analysis of immune-related genes *(NF-κB1*, *IL-1β*, and *TNF-α*) in multiple comparisons between bulls and cows of the Charolais and Holstein breeds (**A**) and between different organs within each experimental animal group (**B**). The data are presented as the means ± SEMs from four independent biological replicates. Stars indicate statistically significant Tukey-adjusted post hoc differences; * (*p* < 0.05), ** (*p* < 0.01), and *** (*p* < 0.001). H_ refers to Holstein cattle, and C_ refers to Charolais cattle. Statistical analyses were performed using ΔCt values within a factorial general linear model including breed, sex, tissue, and their interactions. Relative expression values are presented as fold change for visualization

The expression pattern of *NF-κB1* was significantly affected by tissue (F = 18.323, *p* < 0.001), with a significant sex × tissue interaction (F = 5.775, *p* = 0.002), while breed, sex, breed × sex, breed × tissue, and the three-way interaction were not significant. Post hoc analysis demonstrated limited tissue-specific variation, with significant differences restricted to selected organ comparisons (Fig. 3). Similarly, *IL-1β* expression was significantly influenced by sex (F = 16.027, *p* < 0.001) and tissue (F = 39.531, *p* < 0.001), with a significant sex × tissue interaction (F = 5.765, *p* = 0.002), whereas breed and the remaining interaction terms were not significant. Post hoc comparisons indicated broader tissue-dependent variation than *NF-κB1*, with significant differences across spleen, intestine, lung, and liver, consistent with more pronounced tissue-specific modulation of *IL-1β* expression (Fig. 3). In contrast, *TNF-α* expression was significantly affected only by tissue (F = 10.083, *p* < 0.001), with no significant effects of breed, sex, or interaction terms(Fig. 3).

### Breed-dependent expression levels of B-cell-activating genes in the spleen

**Fig. 4:**
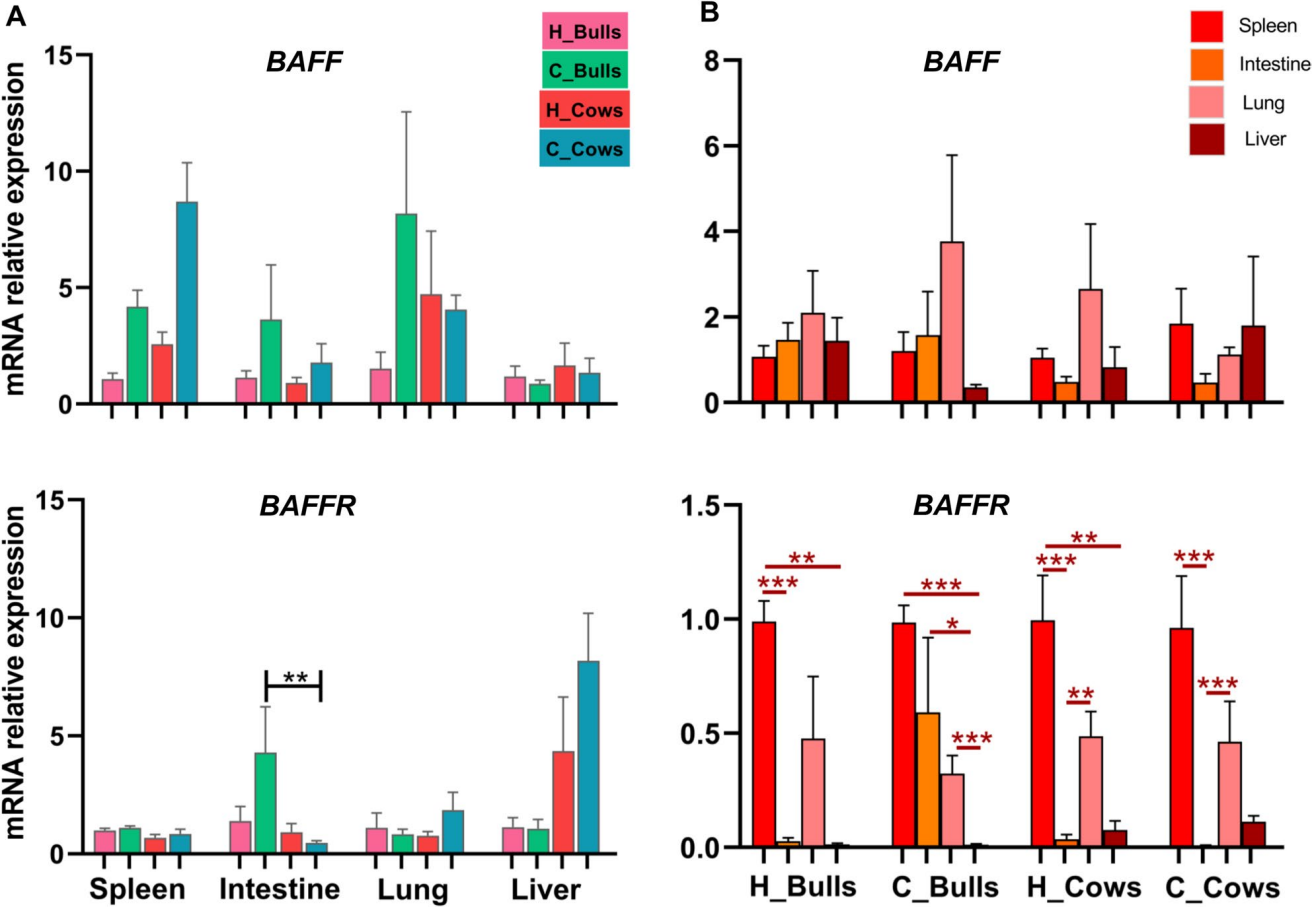
Quantitative PCR analysis of B-cell activation genes (*BAFF* and *BAFFR*) in multiple comparisons between bulls and cows of the Charolais and Holstein breeds (**A**) and between different organs within each experimental animal group (**B**). The data are presented as the means ± SEMs from four independent biological replicates. Stars indicate statistically significant Tukey-adjusted post hoc differences; * (*p* < 0.05), ** (*p* < 0.01), and *** (*p* < 0.001). H_ refers to Holstein cattle, and C_ refers to Charolais cattle. Statistical analyses were performed using ΔCt values within a factorial general linear model including breed, sex, tissue, and their interactions. Relative expression values are presented as fold change for visualization

For *BAFF*, significant main effects of breed (F = 4.929, *p* = 0.031) and tissue (F = 3.189, *p* = 0.032) were observed, whereas sex and all interaction terms were not significant. However, post hoc pairwise comparisons did not reveal significant differences between animal groups or between organs (Fig. 4). *BAFFR* expression was strongly influenced by tissue (F = 54.009, *p* < 0.001), with significant sex × tissue (F = 6.823, *p* < 0.001) and breed × sex × tissue (F = 3.402, *p* = 0.025) interactions, whereas breed, sex, breed × sex, and breed × tissue effects were not significant. Post hoc analysis demonstrated pronounced tissue-dependent variation, with higher *BAFFR* expression generally associated with spleen and lung tissues relative to the intestine and liver in several animal groups (Fig. 4).

### Histological assessment of splenic architecture

**Fig. 5:**
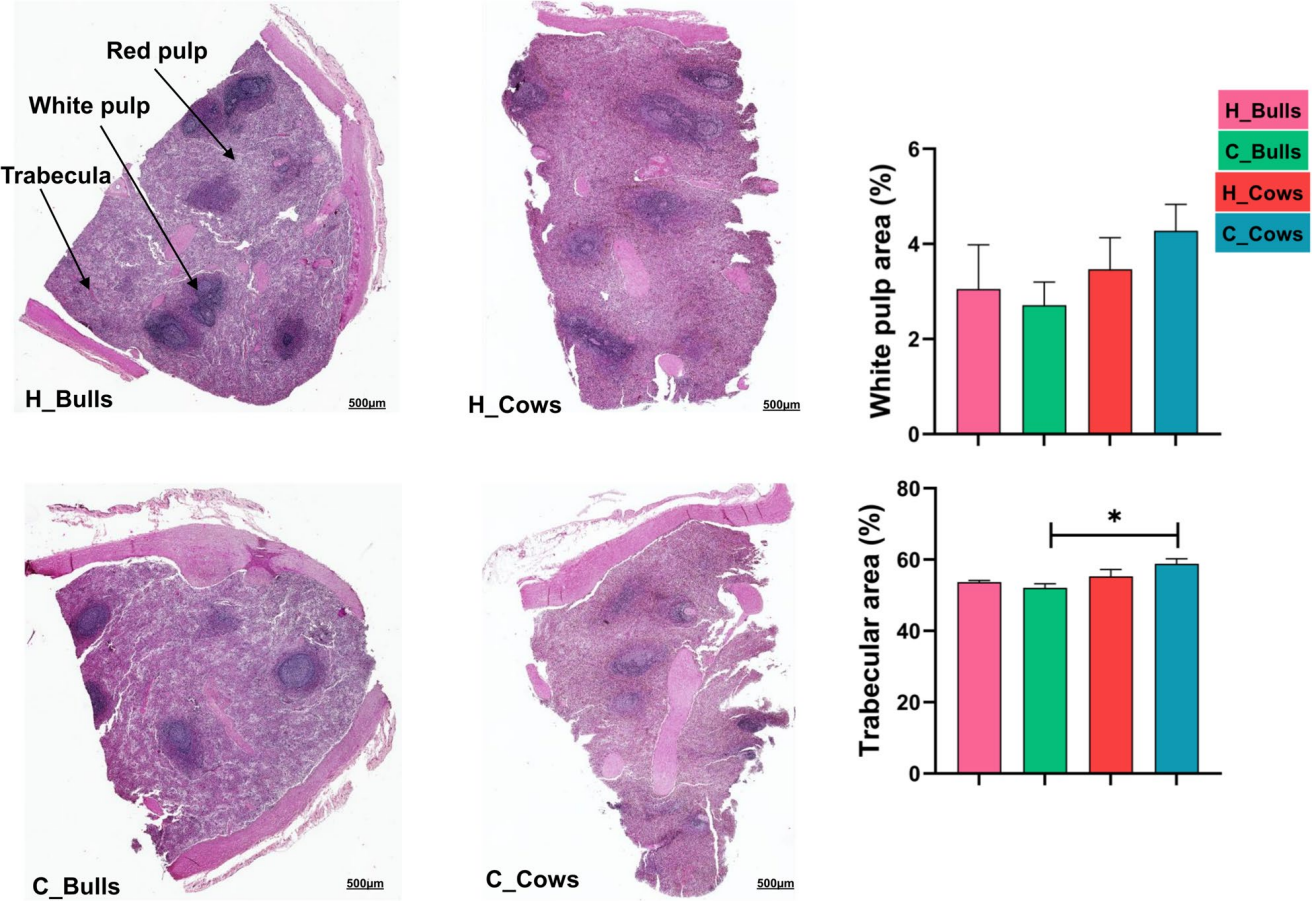
Histological analysis of Holstein and Charolais spleens via hematoxylin and eosin (H&E) staining. H_ refers to Holstein cattle, and C_ refers to Charolais cattle. Quantification of white pulp area expressed as a percentage of total splenic tissue area, based on automated image analysis using identical preprocessing and segmentation parameters across samples. Bars indicate mean ± SEM (*n* = 4 per group), stars indicate statistically significant Tukey-adjusted post hoc differences; * (*p* < 0.05). Analysis revealed sex-associated differences in white pulp proportion, with higher values in females than males in both breeds, while no universal breed-dependent trend was observed. Histological analyses are presented as contextual information and were not used to infer functional differences

Histological analysis was included to verify preserved tissue organization and to exclude gross anatomical differences that could confound transcriptional comparisons. The results revealed preserved and well-organized splenic architecture in all animals, with clearly distinguishable red pulp and white pulp compartments and no overt pathological alterations, indicating preserved tissue architecture across animals. Quantitative histomorphometry analysis revealed variation in white pulp area across sex and breed groups, with higher white pulp proportions observed in females compared with males in both Holstein and Charolais cattle (Fig. 5). Among males, Charolais animals exhibited the lowest white pulp proportions, whereas white pulp area was comparable between breeds in females. In representative sections, Charolais spleens displayed more sharply demarcated white pulp regions and prominent trabecular structures. Consistent with these observations, trabecular prominence showed a tendency to be greater in females than in males, with the highest values observed in Charolais females, while differences between breeds in males were modest (Fig. 5). In addition, qualitative assessment showed a lower frequency of central arterioles within the white pulp in Holstein bulls compared with the other animal groups. Overall, splenic microanatomy appeared preserved across all animals, and variation in compartmentalization was more pronounced between sexes than between breeds.

### In silico structural annotation of a population-prioritized CD14 missense variant

Structural analyses are presented as contextual annotation of coding variation and describe model-based structural differences rather than biological function. A population-prioritized *CD14* missense variant (Asn177Asp) identified from the public genomic dataset, was selected to assess the structural plausibility of coding variation without inferring functional consequences. The substitution is located within the extracellular domain of *CD14*, a region previously described as part of the extracellular domain involved in ligand-associated interactions. Protein stability predictions using DynaMut2 suggested a modest destabilizing effect of the Asn177Asp substitution, with a negative *ΔΔG* value, suggesting reduced thermodynamic stability relative to the wild-type structure. Consistent with this observation, flexibility analysis performed using CABS-flex revealed localized changes in residue-level fluctuations surrounding the mutation site, while global protein dynamics remained largely conserved Table 4.

**Table 4:** Integrated effect of N177D mutation on structure and ligand binding

Parameter	Wild type	N177D	Interpretation
*ΔΔG* (kcal/mol)	—	−1.04	Destabilizing mutation
Mean RMSF (Å)	1.04	1.16	Increased flexibility
Max RMSF (Å)	4.51	8.73	Strong local mobility
Lipid A best Vina (kcal/mol)	−6.7	−6.6	Comparable affinity
Lipid A binding pocket	C5	C1	Pocket relocation
Lipid A cavity volume (Å³)	267	886	Expanded cavity
MPLA Vina (kcal/mol)	−2.5	−2.5	No affinity change

**Fig. 6:**
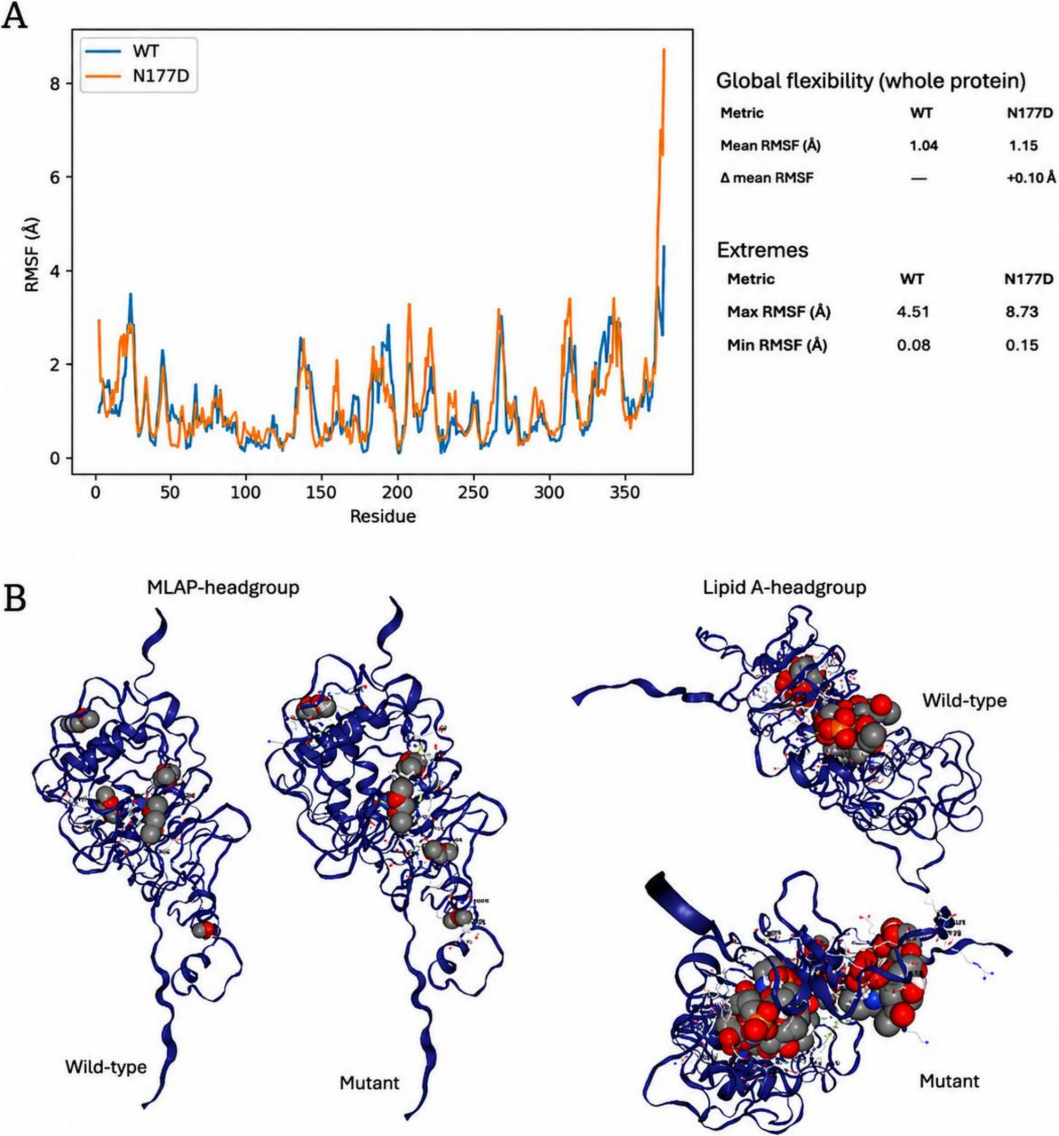
In silico structural evaluation of the population-prioritized *CD14* Asn177Asp missense variant identified from the 1000 Bull Genomes dataset. **A** Residue-level flexibility profiles generated by CABS-flex for wild-type and mutant *CD14* models, showing localized differences in conformational fluctuations near residue 177. **B** Three-dimensional structure of bovine *CD14* highlighting the location of residue 177 within the extracellular domain. The wild-type Asn177 and mutant Asp177 residues are shown as stick representations

To further examine structural features associated with this substitution, blind docking simulations with a lipid A–derived ligand were conducted using a cavity-detection–guided approach. Docking results suggested similar docking scores between the wild-type and mutant *CD14* models. However, differences were observed in the spatial distribution of predicted binding poses and in the usage of surface cavities proximal to residue 177, suggesting localized differences in ligand interaction geometry rather than overall binding strength (Fig. 6). These analyses describe local structural differences associated with the substitution and serve as a contextual annotation of coding variation.

## Discussion

This study presents an integrative genomic analysis combining population-level sequence variation with tissue-specific transcriptional profiling of NF-κB pathway-related genes in cattle. Although the observed variants do not directly establish causality, consistent expression differences were detected across the studied tissues. The tissue-dependent nature of the expression patterns highlights the importance of tissue context when describing NF-κB pathway gene expression. Using population-level sequence data, we prioritized differentiated variants within selected NF-κB pathway-related genes (*CD14*, *TLR4*, *NF-κB1*, *TNF-α*, *IL-1β*, *BAFF*, and *BAFFR*) and examined their tissue-specific transcriptional profiles in Holstein and Charolais cattle.

To address the first aim, the extent of genetic differentiation was quantified via the F_ST_ statistic (Holstein versus Charolais) across the analysed candidate genes to prioritize variants for validation. According to a recent study [35], based on SNP array data from the Holstein and Charolais breeds across various countries, the FST statistic was reported to range from 0.074 to 0.1. The estimates were lower (Table 2), which may partly reflect the use of whole-genome sequence data rather than SNP arrays enriched for common, highly polymorphic markers that are used in other studies. Our results revealed that the *IL-1β* gene presented the highest F_ST_ statistic among the studied genes, indicating comparatively elevated genetic differentiation between the Holstein and Charolais breeds at this genomic location. Furthermore, merging the top F_ST_ values for each gene with the VEP results revealed that 90.55% of the variants were located in introns, which can contribute to regulatory variation affecting transcription [36]. The remaining 9.45% of the determined variants were annotated in exonic gene regions. Selected prioritized coding variants identified from the 1000 Bull Genomes dataset were further examined in the experimentally sequenced validation animals (Table 3) to assess consistency with the population-level prioritization. Although breed-associated differentiation was most evident for the *NF-κB1* variant, the *CD14* and *BAFFR* variants were retained as biologically relevant candidates based on their predicted functional consequences and the broader population-level genomic screening. Not all prioritized public-dataset variants showed breed separation in the limited experimental validation cohort, highlighting the exploratory nature of this integrative design. Furthermore, two of these variants were confirmed to have different allele frequencies in the present study. Specifically, the allele frequencies were confirmed for the *NF-κB1* variant at BTA6:22,225,005 (allele A is segregated in Charolais, whereas in Holstein cattle, it is fixed for reference allele G). The computational evaluation of the *CD14* Asn177Asp variant provides an illustrative example of how sequence variation can be examined in a structural context. The predicted decrease in stability and localized increase in flexibility are consistent with the introduction of a charged residue in place of a neutral polar amino acid, which is consistent with localized structural rearrangements. Importantly, docking simulations did not reveal a substantial difference in predicted ligand binding affinity between the wild-type and mutant *CD14*, indicating that the substitution is unlikely to disrupt ligand recognition dramatically. Instead, differences were observed in docking pose distribution and cavity usage, reflecting variation in local structural context. The docking and flexibility analyses do not account for membrane context, co-receptor interactions, or dynamic immune complex formation involving MD-2 and TLR4. Experimental validation would be required to assess whether the *CD14* Asn177Asp variant is associated with measurable differences under physiological conditions. Within the broader context of this study, the CD14 structural analysis serves as a complementary case study illustrating structural evaluation of prioritized coding variants identified from the population genomic screen rather than as definitive proof of functional impact. Accordingly, the structural analyses are best interpreted as a genomics-oriented annotation layer complementing population differentiation and transcription profiling. Previous cattle genomic studies have linked immune regulatory loci to disease susceptibility through expression-associated mechanisms. For example, integrative expression quantitative trait locus (eQTL) and Mendelian randomization analyses have implicated NF-κB-related signaling pathways in susceptibility to bovine paratuberculosis, supporting the biological plausibility that genomic variation may contribute to differential immune transcriptional regulation in cattle [37, 38]. More broadly, genome-wide transcriptional studies in cattle have demonstrated that host genetic background can influence the expression of immune-related pathways [39]. Although the present study was not designed to establish causal genotype–expression relationships, these observations provide relevant biological context for the breed-associated transcriptional differences observed in the current study.

We investigated the transcription profiles of candidate NF-κB signaling genes that encode receptor (*CD14* and *TLR4*), transcription factor (*NF-κB1*), cytokine (*TNF-α* and *IL-1β*), and B-cell-activating (*BAFF* and *BAFFR*) genes. The *NF-κB1* gene encodes a transcription factor that binds to DNA, resulting in the transcription of numerous genes involved in the immune response [1, 40, 41]. Cytokines, including the *TNF-α* and *IL-1β* genes, are known to be downstream of the NF-κB1 gene. *TNF-α* is a pro-inflammatory cytokine that belongs to the TNF superfamily and regulates several physiological functions in response to numerous immune alterations [42]. The *IL-1β* gene is also a pro-inflammatory cytokine and plays a critical role in defense against different types of infection [43]. Our results revealed tissue-dependent variation in the expression of *NF-κB1*, *IL1β*, and *TNF-α*, although the magnitude and distribution of these differences varied among genes. *IL1β* showed broader tissue-associated variation, whereas *TNF-α* exhibited comparatively limited variation across breeds and sexes. These findings are consistent with the context-dependent activation of canonical NF-κB signaling in immune and peripheral tissues. The NF-κB1 protein is reportedly involved in the development and function of primary and secondary lymphoid tissues, including the spleen [44]. On the other hand, the NF-κB1 protein is involved in canonical and noncanonical pathways, where the latter predominantly targets the p52/RelB NF-κB1 complex, which plays a role in B cells, an adaptive immune cell type, development, and activity [45]. Moreover, it has been reported that NF-κB1 protein plays vital roles in cell fate decisions, survival, and the population of B cells [46], whereas stress can alter leukocyte profiles and immune gene expression in cattle [47]. Notably, *CD14* and *TLR4*, which function as upstream pattern-recognition receptors in canonical NF-κB activation, showed strong tissue-dependent expression patterns. *TLR4* demonstrated a highly consistent tissue-dependent expression profile characterized by significantly lower expression in the intestine, whereas *CD14* showed comparatively elevated expression in liver-associated comparisons, suggesting organ-specific differences in innate immune sensing and receptor-mediated inflammatory signaling.

In this context, mRNA expression analysis was performed for the B-cell-activating genes *BAFF* (a ligand) and *BAFFR* (a receptor), which activate the noncanonical NF-κB pathway. Expression analysis of the noncanonical NF-κB pathway components *BAFF* and *BAFFR* revealed distinct regulatory patterns. *BAFF* expression was influenced by both breed and tissue, suggesting broader transcriptional modulation across animal groups, whereas *BAFFR* exhibited a stronger tissue-dependent profile with significant interaction effects involving sex and tissue. These differences were observed alongside the breed differentiation identified in the sequence data. At the organ level, *BAFFR* expression showed pronounced tissue-specific variation, with relatively higher expression observed in lymphoid and lung tissues compared with liver in several animal groups. The *BAFF* and *BAFFR* genes are TNF family members that function mainly to induce an antibody-mediated immune response through B-cell activation and differentiation [45, 48]. Because B cells preferentially localize within secondary lymphoid tissues such as the spleen, tissue-dependent variation in the expression of these genes is biologically plausible. However, the present findings suggest that regulation of noncanonical NF-κB signaling is not restricted to splenic tissue alone, as substantial variation was also observed across other peripheral tissues. Together with the expression profiles of *CD14*,* TLR4*,* NF-κB1*,* IL1β*, and *TNF-α*, these data support tissue-specific modulation of both canonical and noncanonical NF-κB pathway components in cattle. The spleen, a secondary lymphoid organ, is one of the most essential parts of the mammalian immune system [49]. The splenic pulp is further divided into red pulp and white pulp [50], which have evolved to serve certain immune functions [49]. Histological analysis of the spleen provided complementary contextual information regarding immune organ organization. All animals exhibited preserved splenic architecture, indicating that the observed transcriptional differences are unlikely to be driven by gross pathological alterations. Quantitative assessment revealed that sex-associated variation in white pulp and trabecular organization was more pronounced than breed-associated differences, with females of both breeds showing higher white pulp proportions than males (Fig. 5). While representative sections suggested sharper white pulp demarcation and increased trabecular prominence in Charolais animals, these features were not consistently observed across sexes, indicating the absence of a universal breed-dependent structural pattern. Together, these findings suggest that observed genetic and transcriptional differences were not accompanied by consistent anatomical differences in the spleen. In this study, we integrated population-level genomic data with tissue-specific transcriptional profiling to characterize breed-associated variation in NF-κB signaling-related genes in cattle. The limitations of this study include the modest size of the experimental cohort and the targeted nature of the transcriptional analyses, which focused on selected candidate genes rather than transcriptome-wide profiling. Accordingly, broader population-level inference will require larger independent cohorts and complementary high-throughput functional approaches. An additional limitation is the absence of protein-level validation for the observed transcriptional differences. Direct genotype–expression associations were not assessed because only a subset of expression-profiled animals overlapped with the sequencing validation cohort, and the sample size was insufficient for formal association analysis. Therefore, the present findings should be interpreted as transcriptional associations rather than direct evidence of altered protein abundance or pathway activation.

## Conclusion

This study integrated population-level genomic differentiation with targeted tissue-specific transcriptional profiling of NF-κB pathway genes in Holstein and Charolais cattle. Population-based genomic prioritization identified candidate variants showing breed-associated differentiation, while expression analyses revealed tissue-dependent transcriptional variation across immune-relevant organs. Structural modeling of a population-prioritized CD14 missense variant provided illustrative contextual annotation rather than functional validation. These findings provide descriptive evidence linking genomic differentiation with transcriptional heterogeneity, while larger genotype–expression studies will be required to determine potential causal relationships. 

## Supplementary Information

Supplementary Material 1.

Supplementary Material 2.

Supplementary Material 3.

## Data Availability

The sequencing data generated during the current study are available on ENA under the project accession: PRJEB67743.

## References

[1] Oeckinghaus A, Ghosh S. The NF-kappaB family of transcription factors and its regulation. Cold Spring Harb Perspect Biol. 2009;1:a000034. 10.1101/cshperspect.a000034. 20066092 10.1101/cshperspect.a000034 PMC2773619

[2] Hayden MS, West AP, Ghosh S. NF-kappaB and the immune response. Oncogene. 2006;25:6758–80. 10.1038/sj.onc.1209943. 17072327 10.1038/sj.onc.1209943

[3] Liu T, Zhang L, Joo D, Sun S-C. NF-κB signaling in inflammation. Signal Transduct Target Ther. 2017;2:17023. 10.1038/sigtrans.2017.23. 29158945 10.1038/sigtrans.2017.23 PMC5661633

[4] Zhou Y, Cui C, Ma X, Luo W, Zheng SG, Qiu W. Nuclear Factor κB (NF-κB)-Mediated Inflammation in Multiple Sclerosis. Front Immunol. 2020;11:391. 10.3389/fimmu.2020.00391. 32265906 10.3389/fimmu.2020.00391 PMC7105607

[5] Mallard BA, Leslie KE, Dekkers JC, Hedge R, Bauman M, Stear MJ. Differences in bovine lymphocyte antigen associations between immune responsiveness and risk of disease following intramammary infection with Staphylococcus aureus. J Dairy Sci. 1995;78:1937–44. 10.3168/jds.S0022-0302(95)76819-9. 8550903 10.3168/jds.S0022-0302(95)76819-9

[6] Wilkie B, Mallard B. Selection for high immune response: an alternative approach to animal health maintenance? Vet Immunol Immunopathol. 1999;72:231–5. 10.1016/s0165-2427(99)00136-1. 10614513 10.1016/s0165-2427(99)00136-1

[7] Vignal A, Milan D, SanCristobal M, Eggen A. A review on SNP and other types of molecular markers and their use in animal genetics. Genet Sel Evol. 2002;34:275–305. 10.1186/1297-9686-34-3-275. 12081799 10.1186/1297-9686-34-3-275 PMC2705447

[8] Hill AV. The genomics and genetics of human infectious disease susceptibility. Annu Rev Genomics Hum Genet. 2001;2:373–400. 10.1146/annurev.genom.2.1.373. 11701655 10.1146/annurev.genom.2.1.373

[9] Skevaki C, Pararas M, Kostelidou K, Tsakris A, Routsias JG. Single nucleotide polymorphisms of Toll-like receptors and susceptibility to infectious diseases. Clin Exp Immunol. 2015;180:165–77. 10.1111/cei.12578. 25560985 10.1111/cei.12578 PMC4408151

[10] de Mesquita AQ, Rezende E, Mesquita CSM, de, Da Jardim AJ, Kipnis EAGV. Association of TLR4 polymorphisms with subclinical mastitis in Brazilian holsteins. Braz J Microbiol. 2012;43:692–7. 10.1590/S1517-83822012000200034. 24031881 10.1590/S1517-83822012000200034 PMC3768839

[11] Sharma BS, Mount J, Karrow NA. Functional characterization of a single nucleotide polymorphism in the 5’ UTR region of the bovine toll-like receptor 4 gene. Dev Biol (Basel). 2008;132:331–6. 10.1159/000317179. 18817322 10.1159/000317179

[12] Seabury CM, Seabury PM, Decker JE, Schnabel RD, Taylor JF, Womack JE. Diversity and evolution of 11 innate immune genes in Bos taurus taurus and Bos taurus indicus cattle. Proc Natl Acad Sci U S A. 2010;107:151–6. 10.1073/pnas.0913006107. 20018671 10.1073/pnas.0913006107 PMC2806759

[13] Garbers C, Aparicio-Siegmund S, Rose-John S. The IL-6/gp130/STAT3 signaling axis: recent advances towards specific inhibition. Curr Opin Immunol. 2015;34:75–82. 10.1016/j.coi.2015.02.008. 25749511 10.1016/j.coi.2015.02.008

[14] Zhang Y, Wang X, Jiang Q, Hao H, Ju Z, Yang C, et al. DNA methylation rather than single nucleotide polymorphisms regulates the production of an aberrant splice variant of IL6R in mastitic cows. Cell Stress Chaperones. 2018;23:617–28. 10.1007/s12192-017-0871-0. 29353404 10.1007/s12192-017-0871-0 PMC6045551

[15] Wang X, Zhong J, Gao Y, Ju Z, Huang J. A SNP in intron 8 of CD46 causes a novel transcript associated with mastitis in Holsteins. BMC Genomics. 2014;15:630. 10.1186/1471-2164-15-630. 25070150 10.1186/1471-2164-15-630 PMC4124149

[16] Pant SD, Schenkel FS, Leyva-Baca I, Sharma BS, Karrow NA. Identification of polymorphisms in bovine TLR2 and CARD15,associations between CARD15 polymorphisms and milk somatic cell score in Canadian Holsteins, and functional relevance of SNP c.3020AT. Dev Biol (Basel). 2008;132:247–53. 10.1159/000317167. 18817309 10.1159/000317167

[17] Leyva-Baca I, Schenkel F, Martin J, Karrow NA. Polymorphisms in the 5’ upstream region of the CXCR1 chemokine receptor gene, and their association with somatic cell score in Holstein cattle in Canada. J Dairy Sci. 2008;91:407–17. 10.3168/jds.2007-0142. 18096965 10.3168/jds.2007-0142

[18] Leyva-Baca I, Pighetti G, Karrow NA. Genotype-specific IL8RA gene expression in bovine neutrophils in response to Escherichia coli lipopolysaccharide challenge. Anim Genet. 2008;39:298–300. 10.1111/j.1365-2052.2008.01711.x. 18371126 10.1111/j.1365-2052.2008.01711.x

[19] Mei C, Gui L, Hong J, Raza SHA, Aorigele C, Tian W, et al. Insights into adaption and growth evolution: a comparative genomics study on two distinct cattle breeds from Northern and Southern China. Mol Ther Nucleic Acids. 2021;23:959–67. 10.1016/j.omtn.2020.12.028. 33614243 10.1016/j.omtn.2020.12.028 PMC7868925

[20] Hayes BJ, Daetwyler HD. 1000 Bull Genomes Project to Map Simple and Complex Genetic Traits in Cattle: Applications and Outcomes. Annu Rev Anim Biosci. 2019;7:89–102. 10.1146/annurev-animal-020518-115024. 30508490 10.1146/annurev-animal-020518-115024

[21] Kubes P, Jenne C. Immune Responses in the Liver. Annu Rev Immunol. 2018;36:247–77. 10.1146/annurev-immunol-051116-052415. 29328785 10.1146/annurev-immunol-051116-052415

[22] Bienenstock J. The lung as an immunologic organ. Annu Rev Med. 1984;35:49–62. 10.1146/annurev.me.35.020184.000405. 6372668 10.1146/annurev.me.35.020184.000405

[23] Mowat AM, Agace WW. Regional specialization within the intestinal immune system. Nat Rev Immunol. 2014;14:667–85. 10.1038/nri3738. 25234148 10.1038/nri3738

[24] Lewis SM, Williams A, Eisenbarth SC. Structure and function of the immune system in the spleen. Sci Immunol. 2019. 10.1126/sciimmunol.aau6085. 30824527 10.1126/sciimmunol.aau6085 PMC6495537

[25] Cunningham F, Allen JE, Allen J, Alvarez-Jarreta J, Amode MR, Armean IM, et al. Ensembl 2022. Nucleic Acids Res. 2022;50:D988–95. 10.1093/nar/gkab1049. 34791404 10.1093/nar/gkab1049 PMC8728283

[26] McLaren W, Gil L, Hunt SE, Riat HS, Ritchie GRS, Thormann A, et al. The Ensembl Variant Effect Predictor. Genome Biol. 2016;17:122. 10.1186/s13059-016-0974-4. 27268795 10.1186/s13059-016-0974-4 PMC4893825

[27] Weir BS, Cockerham CC, ESTIMATING F-STATISTICS FOR THE ANALYSIS, OF POPULATION STRUCTURE. Evolution. 1984;38:1358–70. 10.1111/j.1558-5646.1984.tb05657.x. 28563791 10.1111/j.1558-5646.1984.tb05657.x

[28] Danecek P, Auton A, Abecasis G, Albers CA, Banks E, DePristo MA, et al. The variant call format and VCFtools. Bioinformatics. 2011;27:2156–8. 10.1093/bioinformatics/btr330. 21653522 10.1093/bioinformatics/btr330 PMC3137218

[29] Li H, Durbin R. Fast and accurate short read alignment with Burrows-Wheeler transform. Bioinformatics. 2009;25:1754–60. 10.1093/bioinformatics/btp324. 19451168 10.1093/bioinformatics/btp324 PMC2705234

[30] Danecek P, Bonfield JK, Liddle J, Marshall J, Ohan V, Pollard MO et al. Twelve years of SAMtools and BCFtools. Gigascience. 2021. 10.1093/gigascience/giab008 10.1093/gigascience/giab008 PMC7931819 33590861

[31] Chang CC, Chow CC, Tellier LC, Vattikuti S, Purcell SM, Lee JJ. Second-generation PLINK: rising to the challenge of larger and richer datasets. Gigascience. 2015;4:7. 10.1186/s13742-015-0047-8. 25722852 10.1186/s13742-015-0047-8 PMC4342193

[32] R Core Team. R: A Language and Environment for Statistical Computing. Vienna, Austria: R Foundation for Statistical Computing; 2025.

[33] Livak KJ, Schmittgen TD. Analysis of relative gene expression data using real-time quantitative PCR and the 2(-Delta Delta C(T)) Method. Methods. 2001;25:402–8. 10.1006/meth.2001.1262. 11846609 10.1006/meth.2001.1262

[34] Vandesompele J, de Preter K, Pattyn F, Poppe B, van Roy N, de Paepe A, Speleman F. Accurate normalization of real-time quantitative RT-PCR data by geometric averaging of multiple internal control genes. Genome Biol. 2002;3:RESEARCH0034. 10.1186/gb-2002-3-7-research0034. 12184808 10.1186/gb-2002-3-7-research0034 PMC126239

[35] Hall SJG. Genetic Differentiation among Livestock Breeds-Values for Fst. Anim (Basel). 2022. 10.3390/ani12091115. 10.3390/ani12091115 PMC9103131 35565543

[36] Rose AB. Introns as Gene Regulators: A Brick on the Accelerator. Front Genet. 2018;9:672. 10.3389/fgene.2018.00672. 30792737 10.3389/fgene.2018.00672 PMC6374622

[37] van den Berg I, Hayes BJ, Chamberlain AJ, Goddard ME. Overlap between eQTL and QTL associated with production traits and fertility in dairy cattle. BMC Genomics. 2019;20:291. 10.1186/s12864-019-5656-7. 30987590 10.1186/s12864-019-5656-7 PMC6466667

[38] Badia-Bringué G, Canive M, Fernandez-Jimenez N, Lavín JL, Casais R, Blanco-Vázquez C, et al. Summary-data based Mendelian randomization identifies gene expression regulatory polymorphisms associated with bovine paratuberculosis by modulation of the nuclear factor Kappa β (NF-κß)-mediated inflammatory response. BMC Genomics. 2023;24:605. 10.1186/s12864-023-09710-w. 37821814 10.1186/s12864-023-09710-w PMC10568764

[39] Xiang R, MacLeod IM, Daetwyler HD, Jong G, de, O’Connor E, Schrooten C, et al. Genome-wide fine-mapping identifies pleiotropic and functional variants that predict many traits across global cattle populations. Nat Commun. 2021;12:860. 10.1038/s41467-021-21001-0. 33558518 10.1038/s41467-021-21001-0 PMC7870883

[40] Chandel NS, Trzyna WC, McClintock DS, Schumacker PT. Role of oxidants in NF-kappa B activation and TNF-alpha gene transcription induced by hypoxia and endotoxin. J Immunol. 2000;165:1013–21. 10.4049/jimmunol.165.2.1013. 10878378 10.4049/jimmunol.165.2.1013

[41] Oliveira-Nascimento L, Massari P, Wetzler LM. The Role of TLR2 in Infection and Immunity. Front Immunol. 2012;3:79. 10.3389/fimmu.2012.00079. 22566960 10.3389/fimmu.2012.00079 PMC3342043

[42] Kushibiki S. Tumor necrosis factor-α-induced inflammatory responses in cattle. Anim Sci J. 2011;82:504–11. 10.1111/j.1740-0929.2011.00931.x. 21794006 10.1111/j.1740-0929.2011.00931.x

[43] Dinarello CA. Biologic basis for interleukin-1 in disease. Blood. 1996;87:2095–147. 8630372

[44] Burkly L, Hession C, Ogata L, Reilly C, Marconi LA, Olson D, et al. Expression of relB is required for the development of thymic medulla and dendritic cells. Nature. 1995;373:531–6. 10.1038/373531a0. 7845467 10.1038/373531a0

[45] Sun S-C. Non-canonical NF-κB signaling pathway. Cell Res. 2011;21:71–85. 10.1038/cr.2010.177. 21173796 10.1038/cr.2010.177 PMC3193406

[46] Guldenpfennig C, Teixeiro E, Daniels M. NF-kB’s contribution to B cell fate decisions. Front Immunol. 2023;14:1214095. 10.3389/fimmu.2023.1214095. 37533858 10.3389/fimmu.2023.1214095 PMC10391175

[47] O’Loughlin A, McGee M, Waters SM, Doyle S, Earley B. Examination of the bovine leukocyte environment using immunogenetic biomarkers to assess immunocompetence following exposure to weaning stress. BMC Vet Res. 2011;7:45. 10.1186/1746-6148-7-45. 21834971 10.1186/1746-6148-7-45 PMC3177877

[48] Batten M, Groom J, Cachero TG, Qian F, Schneider P, Tschopp J, et al. BAFF mediates survival of peripheral immature B lymphocytes. J Exp Med. 2000;192:1453–66. 10.1084/jem.192.10.1453. 11085747 10.1084/jem.192.10.1453 PMC2193190

[49] Bronte V, Pittet MJ. The spleen in local and systemic regulation of immunity. Immunity. 2013;39:806–18. 10.1016/j.immuni.2013.10.010. 24238338 10.1016/j.immuni.2013.10.010 PMC3912742

[50] Steiniger BS. Human spleen microanatomy: why mice do not suffice. Immunology. 2015;145:334–46. 10.1111/imm.12469. 25827019 10.1111/imm.12469 PMC4479533

