# Supplementary material for: Integrative population genomics and tissue-specific expression profiling in cattle using whole-genome sequence resources
Source: BMC Genomics. 2026 Jul 25;27:638. doi: 10.1186/s12864-026-13218-4 (PMC13401305; doi:10.1186/s12864-026-13218-4)
Supplement: Supplementary file 1 — Supplementary Material 1. [file 12864_2026_13218_MOESM1_ESM.docx]

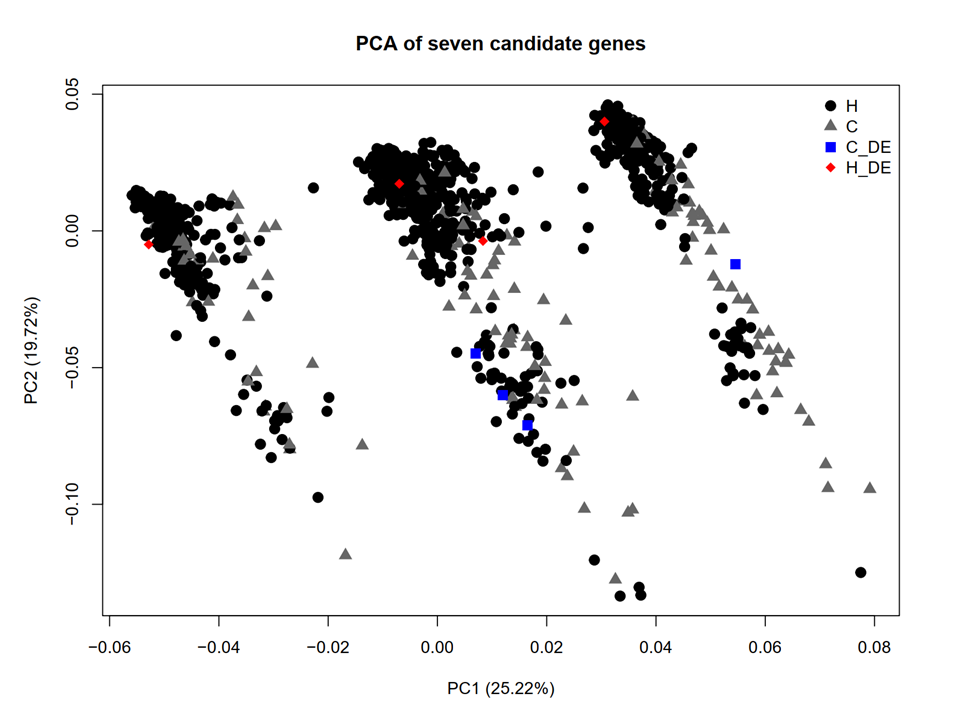


**Supplementary Figure S1.** Principal component analysis of candidate-gene variant profiles from Holstein (H) and Charolais (C) cattle. Principal component analysis was performed using merged variant data from the seven candidate immune genes across the 1000 Bull Genomes reference populations and the experimentally sequenced validation animals. Experimental Holstein (H_DE) and Charolais (C_DE) animals were mapped broadly within the broader distribution of their corresponding breed reference samples in the candidate-gene variant space analyzed.
