# Supplementary material for: Integrative population genomics and tissue-specific expression profiling in cattle using whole-genome sequence resources
Source: BMC Genomics. 2026 Jul 25;27:638. doi: 10.1186/s12864-026-13218-4 (PMC13401305; doi:10.1186/s12864-026-13218-4)
Supplement: Supplementary file 2 — Supplementary Material 2. [file 12864_2026_13218_MOESM2_ESM.docx]

| **Gene Name** | **Symbol** | **Primer sequence** |
| --- | --- | --- |
| CD14 molecule | *CD14* | F:5´-ACACCAACCCGAAGCAGTAT-3'  R:5´-5'-ACCTCCTGTTGTCCACGATA-3' |
| Toll-like receptor 4 | *TLR4* | F:5´-ACCCTTGCGTACAGGTTGTT-3'  R:5´-5'-TAGGCCCTGAAATGTGTCGT-3' |
| Nuclear factor kappa B | *NF-κB1* | F:5´-GCCACTACCAACAGCAGATG-3´  R:5´-AGGTCCCACATAGTTGCAGA-3´ |
| Tumor necrosis factor- alpha | *TNF-α* | F:5´-TAGCCGACATCAACTCTCCG-3´  R:5´-ATGGTGTGGGTGAGGAACAA-3´ |
| Interleukin 1 beta | *IL-1β* | F:5´-AAAATCCCTGGTGCTGGCTA-3'  R:5´-5'-CTTGGGGTAGACTTTGGGGT-3' |
| TNF receptor superfamily member 13B | *BAFF* | F:5´-TCAACAAACCCAGATAACAGGA-3´  R:5´-CCAGCAGCTTTCCATCTTTG-3´ |
| TNF receptor superfamily member 13C | *BAFFR* | F:5´-CATGGCATTGTACCGCATGT-3´  R:5´-TTGTGTCCGACTCTTTGCGA-3´ |
| Glyceryladehyde-3-phosphate dehydrogenase | *GAPDH* | F:5´-CCCAGAATATCATCCCTGCT-3´  R:5´-CTGCTTCACCACCTTCTTGA-3´ |
| beta-2-microglobulin | *B2M* | F:5´-TCCAGCGTCCTCCAAAGATT-3´  R:5´-CCTTGCTGTTGGGAGTGAAC-3´ |
| Tyrosine 3-monooxygenase/tryptophan 5-monooxygenase activation protein zeta | *YWHAZ* | F:5´-CAAAAGACGGAAGGTGCTGA-3´  R:5´-GGTATGCTTGCTGTGACTGG-3´ |

**Supplementary Table 4.** Gene full name and primer list.
